# Organisational and staff-related effects on cultural competence in the hospital setting: a cross-sectional online survey of nursing and medical staff

**DOI:** 10.1186/s12913-022-07947-x

**Published:** 2022-05-14

**Authors:** Liane Schenk, Pia-Theresa Sonntag, Patricia Beck, Zohra Khan, Lisa Peppler, Meryam Schouler-Ocak

**Affiliations:** 1 Charité – Universitätsmedizin Berlin, corporate member of Freie Universität Berlin and Humboldt-Universität zu Berlin, Institute of Medical Sociology and Rehabilitation Science, Charitéplatz 1, 10117 Berlin, Germany; 2 FOM University of Applied Sciences for Economics and Management, Institute for Health and Social Affairs (ifgs), Kruppstr. 86, 45145 Essen, Germany; 3 Charité – Universitätsmedizin Berlin, corporate member of Freie Universität Berlin and Humboldt-Universität zu Berlin, Psychiatrische Universitätsklinik der Charité at St. Hedwig Krankenhaus, Große Hamburger Straße 5-11, 10115 Berlin, Germany

**Keywords:** Cultural diversity, Cultural sensitivity, Professional competence, Cultural competence, Healthcare disparities, Culturally competent care, Inpatient, Quality of healthcare, Organizational culture, Quantitative studies

## Abstract

**Background:**

Cultural competence is considered a core qualification for dealing with socio-cultural diversity and balancing disparities in health care.

**Objectives:**

To explore features supporting and inhibiting cultural competence in the hospital at both organisational and staff levels.

**Design:**

Cross-sectional online survey in the form of a full census from May to November 2018.

**Setting:**

Two organisations that run a total of 22 hospitals in Germany.

**Participants:**

Eight hundred nursing and medical professionals [nurses: *n* = 557; doctors: *n* = 243].

**Methods:**

Using the Short Form Cultural Intelligence SCALE (SFCQ), cultural competence was measured and its relation to potential influencing factors at staff level and organisational level examined, using bivariate (t-Test, one-way ANOVA, Pearson and Spearman correlations) and multivariate (multiple linear regression) approaches. Model 1 examined features at organisational level, Model 2 at individual level and Model 3 included organisational and individual features.

**Results:**

The mean cultural competence measured was 3.49 [min.: 1.3; max.: 5.0]. In the bivariate and isolated multivariate models [Models 1 and 2], factors on both organisational and individual levels were significantly related to the hospital staff’s cultural competence. The multivariate overview [Model 3], however, revealed that individual features at staff level were the statistically relevant predictors. Positive influencing features included staff’s assessment of the importance of cultural competence in their professional context [B: 0.368, 95% confidence interval 0.307; 0.429], participation in competence training [B: 0.193; 95% confidence interval 0.112; 0.276] and having a migration background [B: 0.175; 95% confidence interval 0.074; 0.278], while negative features included length of medical service [B: -0.004; 95% confidence interval -0.007; -0.001].

**Conclusions:**

The development and practice of cultural competence appear to be determined less by organisational features and more on the level of individual actors. In addition to staff development, adequate organisational structures and an economic incentive system are required to promote sociocultural diversity in hospitals.

## Introduction

Global migration reached a peak of an estimated 272 million people in 2019 [1].

The “refugee crisis “ in 2015 gave a fresh boost to migration to Germany, too. Currently, 13.7 million people, 16.7% of the total population of Germany, have personal experience of migration [2]. Nevertheless, being a migrant or having ethnic minority status is linked to unequal access to the health system and sometimes to higher risk of illness [3]. The discussion of ways to effectively counter migrants’ health inequality is still ongoing. Cultural competence is seen as one strategy to rebalance inequalities in nursing and medical care and to promote immigrants’ chances of participation in healthcare [4–8]. Two aspects shown to have a positive impact in this context are a solution-oriented working approach, and empathy and tolerance when dealing with cultural diversity. These factors are reflected in improved treatment quality and patient satisfaction, as well as economic efficiency [9–14]. Cultural competence also has the potential to increase healthcare staff’s professional satisfaction and to protect them from perceived time pressure, stress, sleep problems or burnout [15–17].

Pluralisation processes in the health care system are accelerated not only by its users, but also by the employment of (post-)migrant healthcare staff. According to OECD figures, the number of trained healthcare staff in almost all European countries increased in the last decade, yet more are still needed [18]. The WHO predicts a lack of about 14.5 million trained nursing staff by 2030 [19]. One reaction is to recruit trained nurses from abroad [20]. In addition, German policy is to integrate refugees into the healthcare professions [21].

Consequently, healthcare is experiencing more multicultural teams of nurses and doctors and increasing cultural diversity of patients. In other words, immigration impacts the nursing and medical care field in a variety of ways. This diversity can be a challenge that particularly affects communication when actors have different sociocultural backgrounds.

### The concept of cultural competence

Communication is based on internalised patterns of action and interpretation that people acquire during their lives. In our context, the key agent of socialisation is the cultural system ‘medicine’ which has differing cultural and social features in each country. This results in a range of ideas about illness and health, concepts of healthcare and healing, and specific notions of the hierarchies, skills and fields of responsibility of the nursing and medical professions. The fewer experiences interacting persons have in common, the more difficult it is to correctly grasp, much less intuitively interpret, the intention behind others’ words, gestures and emotions. Although interactions are therefore seldom conflict-free as diversity increases, little attention has been paid so far—in Europe—to sociocultural and migration-related aspects in care or organisational concepts [22–29].

Cultural competence is seen as a potential tool for shaping effective, appropriate communication and interaction, regardless of the participants’ sociocultural background [30, 31]. “Cultural competence” is usually described as a multidimensional set of cognitive orientations, culture-related knowledge, skills, sensitivities and attitudes [32, 33]. In this context, (self-)critical reflection is considered particularly important; this includes among other things the individual’s ties to their cultural location, internalised prejudices and value hierarchies.

### Factors that influence cultural competence

It is generally accepted that cultural competence can be learnt [34, 35]. Several literature reviews confirm that coaching is an effective factor in strengthening all, or at least some, dimensions of cultural competence [36–41]. It is less clear how sociodemographic factors such as age, educational level, country of origin, religion and ethnicity are related to cultural competence. While a number of studies show no links [42, 43], several indicate age as an influencing factor [44, 45]. Almutairi et al. (2017) attributed this to the accumulated professional experience of the nurses examined that had a positive effect on their cultural competence, while also showing that cultural competence varied in relation to the nurses’ country of origin [44]. Finally, a Swiss study indicated that doctors have more cultural competence than nursing staff [46].

The analyses in these studies focused primarily on individual factors at staff level. Looking at actions intended to promote cultural competence, they also focused on strategies that aim at staff development and training [35, 47, 48]. Organisational factors (e.g. organisational readiness and commitment, audit and quality improvement approaches, workforce diversity, diversity climate) intended as innovations to promote cultural competence in the healthcare system were measured for their effectiveness in relation to patient-oriented outcomes or at system level [47, 49]; the studies reveal no overall impact. The direct effect of these factors on healthcare staff’s cultural competence was not the focus of this research, however.

### Logic model and study aim

Our concept starts with the assumption that while an organisation‘s institutional framework does not determine its members’ actions, it does influence them. To that extent, organisations can strengthen or inhibit active cultural competence. This article examines how both staff-related and institution-related factors are linked to the cultural competence of healthcare staff, both nurses and doctors, in hospital.

## Methods

### Data collection

Data collection lasted from May to November 2018. Active healthcare workers in 22 hospitals run by two organisations (Organisations A and B) were invited by email to participate in an online survey via a personalised link (Organisation A: approx. 2,500 doctors, approx. 4,000 nursing staff; Organisation B: approx. 1,200 doctors, approx. 5,800 nursing staff). Because some potential respondent groups did not have their own email accounts, they received their invitations, including a QR code, sent with the pay slip. The survey was administered using the EFS software Unipark. The data collected were saved on an external server protected by QuestBack, a Unipark provider. In order to increase the response rate, we sent reminders and an additional survey was carried out later among the doctors in organisation B.

### Measuring “cultural competence”, the target variable

To measure cultural competence, a multidimensional construct, we applied Thomas et al.’s short scale (Short Form Cultural Intelligence SCALE, SFCQ) which provided evidence for construct-related and criterion-related validity and is internet-enabled [50]. This instrument already existed in five languages and was translated from the English original into German (Cronbachs α = 0.87, total value). Several scientists worked in parallel to translate the text and the translation was finalised in a joint sitting [51]. The instrument includes ten items: two measure cultural knowledge, five measure cultural skills and three measure cultural metacognition. Higher scores indicate greater cultural competence. The arithmetic mean was determined across all ten items; cases with two or more missing items (*n* = 6) were excluded. Where one item was missing (*n* = 14), the arithmetic mean of nine items was determined.

### Independent variables at the individual level

Independent variables at individual level were sociodemographic factors and features of respondents’ professional biographies: gender, migration background, occupational category [nursing, medicine], duration of career and respondents’ assessment of how well their training prepared them for working with immigrant patients [well, fairly well, fairly poorly, poorly] and the importance assigned to cultural competence in the professional context [very important, important, slightly important, not important]. Finally, respondents were asked whether they had completed a training course on cultural competence. Data on respondents’ migration background was based on the country of (the parents’) birth and differentiated between whether one or both parents are immigrants [52]. Respondents born in Germany but one of whose parents were born abroad have a “migration background on one side”. Respondents who are themselves immigrants and/or both parents are immigrants have a “migration background on both sides”.

### Independent variables at organisational level

Institutional factors were registered by differentiating between the two organisations [A and B] that run the hospitals, the (estimated) proportion of staff and patients with migration backgrounds on each ward, and regular employer encouragement to take part in intercultural coaching. The latter item was dichotomised by combining the options “don’t know” and “no”.

### Statistical analyses

SPSS Statistics 25 was used to carry out the statistical analysis, including mean comparison tests (t-test, one-way ANOVA), correlation analysis (Pearson, Spearman) and multiple regression in which potential influencing factors were related to cultural competence. Three models were calculated in total: first, effects on the organisational level were examined; second, effects at individual level and third, all variables were examined in a joint model. The model was tested for multicollinearity. We set the significance level for all analyses to α = 0.05.

## Results

### Institution-related and staff-related sample characteristics

A total of 800 active hospital staff participated in the online survey, a response rate of about 6%. Table 1 shows the main characteristics of the organisational and staff samples and how they relate to cultural competence. In the bivariate test, both organisational features (organisation, provision and encouragement of coaching on intercultural topics, proportion of staff and patients with migration background) and individual features (occupational category, migration background, mother tongue, relevance, preparation through training, participation in intercultural competence coaching, career duration) show a significant correlation with the degree of cultural competence.

**Table 1:** Sample composition, bivariate relationship to cultural competence

**Characteristics at organisational level**	**n**	**%**	**Significance**
**Organisation**			
Organisation A	271	33.9	
Organisation B	529	66.1	
Total	800	100,0	.014^a^
**Coaching offer for intercultural competence**			
(*n* = 793)			
yes	339	42.7	.001^a^
**Regular motivation to participate in intercultural coaching**			
(*n* = 789)			
yes	146	18.5	.002^a^
**Region**			
Rural	34	4.3	
Urban	765	95.7	
Total	799	100.0	.412^a^
	Mean	SD	
**Staff share with a migration background (%)**			
(*n* = 800)	22.1	17.48	.002^c^
Min	0		
Max	85		
**Patient share with a migration background (%)**			
(*n* = 800)	30.45	20.51	< .001^c^
Min	0		
Max	100		
**Characteristics at individual level**	**n**	**%**	**Significance**
**Gender**			
Male	241	31.0	
Female	536	69.0	
Total	777	100.0	.433^a^
**Profession**			
Nursing	557	69.6	
Medicine	243	30.4	
Total	800	100.0	.022^a^
**Migration background**			
None	621	78.2	
On one side	56	7.1	
On both sides	117	14.7	
Total	794	100.0	< .001^b^
**Native language**			
German	718	89.9	
Other	82	10.3	
Total	800	100.0	< .001^a^
**Relevance assigned to cultural competence**			
Very important	351	44.1	
Important	385	48.4	
Slightly important /not important	60	7.5	
Total	796	100.0	< .001^d^
**Preparation by professional training**			
Well	49	6.2	
Fairly well	169	21.3	
Fairly poorly	423	53.2	
Poorly	154	19.4	
Total	795	100.0	< .001^d^
**Participated in coaching on cultural competence**			
Yes	194	24.5	
No/ don’t know	599	75.5	
Total	793	100,0	< .001^a^
	Mean	SD	
**Duration of career (years)**			
(*n* = 796)	16.9	12.02	< .001^c^
Min	0		
Max	48		

^a^T-Test

^b^Simple ANOVA

^c^Pearson

^d^Spearman

Significance level α = 0.05

The average cultural competence measured is 3.49. The sub-dimension Cultural Metacognition has a statistically significantly lower mean value than that of Cultural Skills (Table 2). However, this is not relevant for the following analyses, since the Short-Scale SFCQ was designed as a reflexive model in which the three sub-dimensions are not compensatory, but in which cultural competence is reflected as a latent construct in all three sub-dimensions [50].

**Table 2:** Cultural competence of the survey participants

	Minimum	**Mean—CI (95%)**	Median	Maximum	SD
Cultural intelligence (*n* = 794)	1.3	[3.447–3.525]	3.50	5.0	.56
Cultural knowledge (*n* = 797)	1.0	[3.434–3.539]	3.50	5.0	.75
Cultural skills (*n* = 791)	1.2	[3.502–3.587]	3.60	5.0	.60
Cultural metacognition (*n* = 781)	1.0	[3.338–3.438]	3.33	5.0	.70

### Multiple regression models

Table 3 shows the results of the three regression models. The regressors included on the organisational level (Model 1) explain 3.4% of the variance of the target variable “cultural competence”; the regressors on the personnel level (Model 2) explain 29.9% of this variance and those on both organisational and individual levels (Model 3) explain 29.7%.

**Table 3:** Multivariate correlation between cultural competence of hospital staff and characteristics at organisational and individual level

	**Model 1: organisational level**				**Model 2: individual level**				**Model 3: total (organisational + individual)**			
**Variable**	B	β	p	95% CI	B	β	p	95% CI	B	β	p	95% CI
Organisation (B)	-.073	-.061	.095	[-.154, .014]					.009	-.007	.835	[-.068, .086]
Immigrated staff share	.002	.068	.086	[.000, .005]					.001	.023	.505	[-.001, .003]
Immigrated patient share	**.003**	**.102**	.012	**[.001, .005]**					.001	.035	.327	[-.001, .003]
Motivation for coaching (Yes)	**.145**	**.101**	.004	**[.039, .242]**					.021	.015	.656	[-.079, .118]
Profession (medicine)					.022	.019	.565	[-.054, .100]	.031	.026	.461	[-.048, .115]
Gender (female)					.052	.044	.159	[-.021, .122]	.046	.039	.216	[-.028, .120]
Duration of career					**-.004**	**-.092**	.006	**[-.007, -.001]**	**-.004**	**-.083**	.015	**[-.007, -.001]**
Relevance for cultural competence					**.373**	**.415**	< .001	**[.313, .434]**	**.368**	**.410**	< .001	**[.307, .429]**
Preparation by vocational training					**.111**	**.157**	< .001	**[.064, .156]**	**-.107**	**-.151**	< .001	**[.060, .152]**
Participated in coaching					**.204**	**.159**	< .001	**[.127, .278]**	**.193**	**.151**	< .001	**[.112, .276]**
Migration background on one side					.097	.045	.147	[-.040, .232]	.092	.043	.178	[-.044, .276]
Migration background on both sides					**.183**	**.118**	< .001	**[.083, .282]**	**.175**	**.113**	.001	**[.074, .278]**
Constant	3.374		< .001	[3.267, 3.478]	4.366		< .001	[4.170, 4.563]	4.290		< .001	[4.062, 4.506]
N	786				758				755			
R2	.039				.307				.308			
F	7.984				41.506				27.521			
CORR.R2	.034				.299				.297			

Dependent variable: cultural competence; unstandardised coefficients B, standardised coefficients β

*CI* Confidence interval

#### Model 1: predictors at organisational level

Two factors with a significant positive effect on the cultural competence of hospital staff are estimated proportion of patients with a migration background and employer encourages staff to participate in coaching on intercultural topics. As the proportion of patients with a migration background grows, respondents’ cultural competence also increases, though only very slightly, by 0.003 points. The greatest effect on respondents’ cultural competence at organisational level was achieved by regularly encouraging staff to participate in relevant training.

#### Model 2: predictors at individual level

Respondents’ occupational category or gender is not significantly linked to their cultural competence. Factors positively associated with cultural competence are staff’s assessment that cultural competence is very important in the professional context and that their training prepared them well for working with migrant patients. Participation in relevant coaching courses also has a positive effect on the competence measured. The cultural competence of people who had attended such coaching was 0.204 points higher than that of respondents who had not. There is also a significant difference in cultural competence between respondents with a migration background on both sides and those with no migration background. Staff with a migration background on both sides show cultural competence that is 0.183 points higher than those with no migration background. No significant effect could be seen in people with a migration background on one side. Career duration is negatively associated with cultural competence, which sinks very slightly, but still significantly, by 0.004 points per career year. The strongest effect on the individual level, i.e., hospital staff, reflects how relevant they consider cultural competence to be.

#### Model 3: institutional and person-related predictors

If factors on the organisational and individual levels are examined simultaneously in one model, only the staff-related effects are significant. The largest effect is still produced by the importance assigned to cultural competence, followed by participation in coaching and a migration background on both sides. Professional training and career duration also have significant influence.

## Discussion

Sociocultural diversity is a typical feature of the everyday medical and nursing context in hospital. This creates challenges both in dealing with patients and where staff cooperate in a multicultural environment. The initial thesis behind our analysis was that in addition to individual skills at staff level, the institutional context can promote or inhibit cultural competence in action. Therefore the analytical viewpoint includes both individual and institution-related factors.

First, our findings show that cultural competence in action seems to increase with everyday practice in an intercultural context – the proportion of immigrant patients reported in the hospitals we examined indicates this. Equally, the institutional context has a positive effect if the organisation is aware of the importance of intercultural care and, for example, actively encourages its staff to participate in relevant coaching. In the overview of the influencing factors analysed, however, it is the individual resources that produce an effect on cultural competence. In particular, the significance staff allocate to this topic in professional activity produces a positive effect; actually attending relevant coaching is also beneficial. A migration background on both sides and intercultural content in professional training are also associated with greater cultural competence. Longer duration of respondents’ professional careers had a negative impact. The development and practice of cultural competence thus appears to be not so much anchored in the institutional structure as linked to the level of individual actors.

### Factors at organisational level

A staff development strategy focused on cultural diversity has been discussed as an effective institutional instrument for driving cultural competence development [47, 49]. Our isolated examination of organisational factors clearly showed that cultural competence is boosted when the proportion of staff with a migration background increases, even though this effect was slightly below a statistically significant level. This indicates that working in culturally diverse teams is linked to increased demands for culturally competent behaviour. The proportion of patients with a migration background had a greater influence – intercultural care practice may be concomitant with more pressure to improve qualifications. The greatest effect, however, was seen when the institution encourages staff to participate in intercultural coaching; we assess this as indicating a management that actively promotes the development of relevant skills. Studies that identify diversity-sensitive organisational development as the institutional framework for cultural competence in practice point to the same conclusion. This includes factors such as a diversity-sensitive organisational climate, defining cultural competence development as a mark of quality, and coordinated controlling [53, 54].

### Factors at individual level

The positive relationship between staff members’ participation in intercultural coaching and their level of cultural competence tallies with the findings of previous studies. For example, several systematic reviews confirm that coaching increases healthcare workers’ cultural competence [48]. This applies to both intercultural knowledge and culture-sensitive characteristics and attitudes. Despite intercultural education, nevertheless, an in-depth understanding of cross-cultural healthcare (e.g., power imbalances, biases, and self-reflexivity) often seems to be lacking [55].

In their study of doctors and psychotherapists in training, Bernhard et al. (2015) determined that both cultural competence training and a migration background had a significant influence on some dimensions of cultural competence. The results of this online survey confirm that a migration background on both sides has positive effects for the respondents. In particular, potential and resources such as speaking more than one language, specific cultural knowledge and the experience of moving in different sociocultural contexts seem to have a beneficial impact on culture-sensitive patient care. Staff with migration backgrounds also more often take on the role of cultural mediators, interpreters or people of trust in their everyday work context.

Unlike the findings of a Swiss study [46], our survey showed no indications of differences between nurses and doctors related to their professional category.

While some studies show that cultural competence increases with age and professional experience, [44, 45] we observed the opposite effect. We suspect that a generational effect lies behind the negative association between career duration and the level of cultural competence: on the one hand, younger staff may benefit from changes to training content, which increasingly takes culturally sensitive and migration-related topics into account, though not always to the same extent. The effect of career duration still shows up, however, regardless of how their training was oriented, as we were able to show. On the other hand, intercultural contacts both at work and in private life may be taken more for granted by younger staff members and socialisation in contexts of diversity may be more common. Studies show that cultural competence increases with the frequency of intercultural encounters in private and professional contexts [10, 56, 57]. Topics like cultural competence that are relatively new in Germany may therefore be less accessible to older staff members. Finally, however, another explanation could be that where working routines become ossified, cultural competence that may initially have been present may gradually fade.

### Strengths and limitations

The significance of our data is limited by restricted controllability of the survey conditions. The links to the survey were passed on via mediators in each institution for data protection reasons. At the same time, however, coverage problems were reduced because the base population was known and a lack of internet access at the place of work could be compensated by the QR code on the payslip. Online surveys are generally considered to be less successful in motivating respondents to participate, a problem reinforced by the lack of time available during everyday hospital work. This is reflected in the low response rate. In Organisation A the nurses were harder to reach; in Organisation B the doctors were less accessible. The sample nevertheless adequately reflects the overall distribution of the professional categories. Further, we cannot exclude the possibility that respondents who were motivated to take part were those who were more interested in the topic anyway and therefore have more cultural competence. Moreover, information on how to correctly interpret our finding that cultural competence decreases with career length can only be provided by a longitudinal study. Data on organisation-related characteristics were collected through a self-report survey. In order to ensure anonymity, it was not possible to assign survey participants to a particular hospital. Nevertheless, the decisive aspect for the actions of professional actors is how they perceive their organisation and its services. Finally, we subsumed several hospitals in two units run under the auspices of Organisations A and B. This leads to a simplification because individual hospitals run by one organisation may still differ. However, the hospitals present themselves as being linked by a common philosophy which includes addressing sociocultural diversity.

## Conclusion

On balance, our findings suggest that culturally competent action is not promoted systematically through the organisational contexts but is primarily supported by the staff’s individual resources. As diversity increases, in-patient care institutions are coming under increasing pressure to change. This includes promoting organisational structures and an organisational culture that accepts apparent cultural idiosyncrasies as variations in ways of thinking and acting that anchor intergenerational and cultural learning and encourage their staff to consistently apply the cultural competence they have gained. Last but not least, this requires economic incentive systems that reward culturally competent care. In order to optimise care quality in the long term in the context of sociocultural diversity and to ensure the satisfaction of both patients and staff, the process of intercultural opening must include structural development on the organisational level, alongside personnel development.

## Data Availability

The datasets generated and analysed during the current study are not publicly available due to data protection concerns, particularly on the part of the participating hospital organisations, but are available from the corresponding author on reasonable request.

## Abbreviations

OECD: Organisation for Economic Co-operation and Development
SFCQ: Short Form Cultural Intelligence SCALE
WHO: World Health Organization
